# Low expression of LOC285194 is associated with poor prognosis in colorectal cancer

**DOI:** 10.1186/1479-5876-11-122

**Published:** 2013-05-16

**Authors:** Peng Qi, Mi-die Xu, Shu-juan Ni, Dan Huang, Ping Wei, Cong Tan, Xiao-yan Zhou, Xiang Du

**Affiliations:** 1 Department of Pathology, Fudan University Shanghai Cancer Center, Shanghai, 200032, China; 2 Department of Oncology, Shanghai Medical College, Fudan University, Shanghai, 200032, China; 3 Institute of Pathology, Fudan University, Shanghai, 200032, China; 4 Institutes of Biomedical Sciences, Fudan University, Shanghai, 200032, China

**Keywords:** Colorectal cancer, Long non-coding RNAs, LOC285194, Survival

## Abstract

**Background:**

The long non-coding RNAs (lncRNAs) study has gradually become one of the hot topics in the field of RNA biology. One lncRNA which has attracted attention is LOC285194, a lncRNA demonstrated the potential tumor-suppressor role in osteosarcoma. The aim of this study was to examine the expression of LOC285194 in colorectal cancer (CRC) patients and to investigate the relationship between this lncRNA levels and existing clinicopathologic parameters and patient survival.

**Methods:**

The expression of LOC285194 was detected by quantitative real-time polymerase chain reaction in pairs of tumorous and adjacent normal tissues of 81 colorectal cancer patients with a follow-up of 5 years, as well as in three colorectal cancer cell lines and normal intestinal mucous cell line. Then, we analyzed the potential relationship between this lncRNA levels in tumor tissues and existing clinicopathological features of CRC, and clinical outcome.

**Results:**

The relative expression levels of LOC285194 was significantly lower in tumor tissues (*p* < 0.001) and colorectal cancer cell lines compared with adjacent normal tissues and normal intestinal mucous cell line. In addition, low expression of LOC285194 was correlated with larger tumor size (*p* = 0.015), higher tumor stage (*p* = 0.034), and more distant metastasis (*p* = 0.046). Kaplan-Meier analysis indicated that patients with low LOC285194 expression had a poor disease free survival (*p* = 0.010). Moreover, multivariate analysis showed that decreased expression of LOC285194 was an independent predictor of disease-specific survival.

**Conclusion:**

Our data indicate that LOC285194 might be a novel prognostic indicator in colorectal cancer and may be a potential target for diagnosis and gene therapy.

## Background

Colorectal cancer (CRC) is the third most prevalent cancer and the second most common cause of cancer-related death in the world, with an annual death rate exceeding 608,700 according to the National Cancer Institute [1]. Most cases of CRC arise sporadically and usually progress from benign polyp to malignant adenocarcinoma and distant metastasis. For accurate diagnosis and adequate treatment of CRC, identification and understanding of the molecules responsible for cancer progression are critical.

Except for about 2% protein-coding genes, the vast majority of the human genomes are non-coding RNAs (ncRNAs) [2], implicating that ncRNAs could play significant regulatory roles in complex organisms. The function and clinical significance of short regulatory ncRNAs, such as microRNAs (miRNAs) and small-interfering RNAs (siRNAs) were elucidated first and then, long ncRNAs (lncRNAs) were reported more recently. LncRNAs have been shown to be spliced, polyadenylated, and developmentally regulated in eukaryotes, including antisense, intergenic transcripts and epigenetic regulators. Many studies strongly suggested that deregulated expression of lncRNAs is closely correlated with the diversity of multigenetic diseases [3]. In addition, recent reports showed that some lncRNAs exhibit distinct gene expression patterns in solid tumors and leukemias [4-8]. Functional lncRNAs can be used for cancer diagnosis and prognosis, and serve as potential therapeutic targets, we consider them as a new cancer diagnostic and therapeutic gold mine in the future [9].

The LOC285194 gene is 2105 nt in length, located in chr3q13.31, consisting of four exons. LOC285194 is a lncRNA that showed loss of expression in primary osteosarcoma samples and cell lines. In addition, depletion of LOC285194 attributed to proliferation of normal osteoblasts through regulation of apoptotic and cell cycle transcripts and VEGF/VEGFR1. Moreover, genetic deletions of LOC285194 were associated with poor survival of osteosarcoma patients [10]. These data demonstrated the potential tumor-suppressor role of LOC285194 in osteosarcoma; however, the relationship between expression of LOC285194 and CRC development and/or progression remains unclear.

In the current study, we clarified the clinical significance of LOC285194 expression in CRC. The primary aim of this study was to investigate whether LOC285194 is detectable and altered in colorectal cancer tissues or cell lines compared with adjacent normal tissues or normal cell line. Then, a potential relationship between this lncRNA levels in tumor tissues and existing clinicopathological features of CRC, such as tumor size, location, histologic stage, depth of invasion, the status of lymphatic metastasis, venous invasion, nervous invasion, distant metastasis and disease-specific survival (DSS), was investigated.

## Methods

### Cell lines, tissue samples and clinical data collection

A total of 81 patients analyzed in this study underwent resection of the primary CRC at Fudan University Shanghai Cancer Center. The diagnosis of CRC was histopathologically confirmed. No patient received preoperative treatment. Resected tissue samples were immediately frozen in liquid nitrogen, and stored at -80°C until RNA extraction. The data collected on all subjects include age, gender, DSS and CRC features such as tumor size, location, histologic stage, depth of invasion, the status of lymphatic metastasis, venous invasion, nervous invasion, and distant metastasis. Clinical stage of CRC was evaluated on the basis of the TNM classification system [11]. Patient follow-up was performed every 2–3 months during the first year after surgery and 3–6 months thereafter until November 30, 2012. All patients had completed follow-up. The DSS was defined as the length of time between the surgery and death specifically from the cancer. During follow-up, 20 patients died of CRC. This study was approved by The Clinical Research Ethics Committee of Fudan University Shanghai Cancer Center, and informed consent was obtained from participants for the use of their tissues in this study.

The human colorectal cancer cell lines, including CaCO-2, HCT8, LoVo and human normal intestinal mucous cell line CCC-HIE-2 were obtained from the American Type Culture Collection (Manassas, VA, USA). All cell lines were maintained routinely in Dulbecco’s modified Eagle’s medium supplemented with 10% fetal bovine serum and 2 mM L-glutamine (Invitrogen, Carlsbad, CA) and were grown at 37°C in a 10% CO_2_ atmosphere.

### RNA preparation, reverse transcription, and quantitative real-time PCR

Total RNAs were extracted from tumorous and adjacent normal tissues using Trizol (Invitrogen, Carlsbad, CA, USA) following the manufacturer’s protocol. RT and qPCR kits were used to evaluate expression of LOC285194 from tissue samples. The 20 μL RT reactions were performed using a PrimeScript® RT reagent Kit (Takara, Dalian, China) and incubated for 30 min at 37°C, 5 s at 85°C, and then maintained at 4°C. For real-time PCR, 1 μL diluted RT products were mixed with 10 μL of 2 × SYBR® Premix Ex Taq^™^ (Takara, Dalian, China), 0.6 μL forward and reverse primers (10 μM), and 8.4 μL Nuclease-free water in a final volume of 20 μL according to manufacturer instructions. The primers used in this study were 5’-TGTGCCTGTTTGACCTCTGA-3’ (forward) and 5’-AGGAAGGATAAAAGACCGACCA-3’ (reverse). All reactions were run on the Eppendorf Mastercycler EP Gradient S (Eppendorf, Germany) using the following conditions: 95°C for 30 s, followed by 40 cycles at 95°C for 5 s, and 60°C for 30 s. Real-time PCR was done in triplicate, including no-template controls. Amplification of the appropriate product was confirmed by melting curve analysis following amplification. Relative expression of LOC285194 was calculated using the comparative cycle threshold (CT) (2^-ΔΔCT^) method with glyceraldehyde-3-phosphate dehydrogenase (GAPDH) as the endogenous control to normalize the data.

### Statistical analysis

All statistical analyses were performed using SPSS 20.0 software (IBM, SPSS, Chicago, IL, USA). The significance of differences between groups was estimated by Student’s *t*-test, *χ*^2^ test or Wilcoxon test, as appropriate. DSS rates were calculated by the Kaplan-Meier method with the log-rank test applied for comparison. Variables with a value of *p* < 0.05 in univariate analysis were used in subsequent multivariate analysis on the basis of Cox proportional hazards model. Two-sided *p*-values were calculated, and a probability level of 0.05 was chosen for statistical significance.

## Results

### Expression of LOC285194 in tissues and cell lines

**Figure 1:**
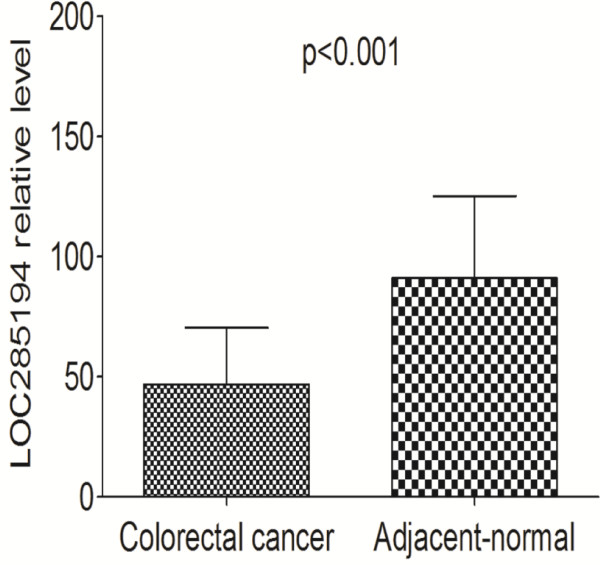
**LOC285194 expression levels assessed by quantitative real-time PCR in cancerous tissue and adjacent normal mucosa.** The LOC285194 expression levels were normalized to GAPDH. LOC285194 expression was significantly lower in cancerous tissues compared with noncancerous tissues. Data are means ± SEM (p < 0.001).

The first goal of the present study was to investigate whether LOC285194 is detectable and altered in colorectal cancer tissues compared with adjacent normal tissues. Using RNA isolated from tissues, we performed RT-qPCR to detect the expression levels of LOC285194. Using GAPDH as normalization control, LOC285194 expression was significantly lower in tumor tissues compared with adjacent normal tissues (p < 0.001; Figure 1).

**Figure 2:**
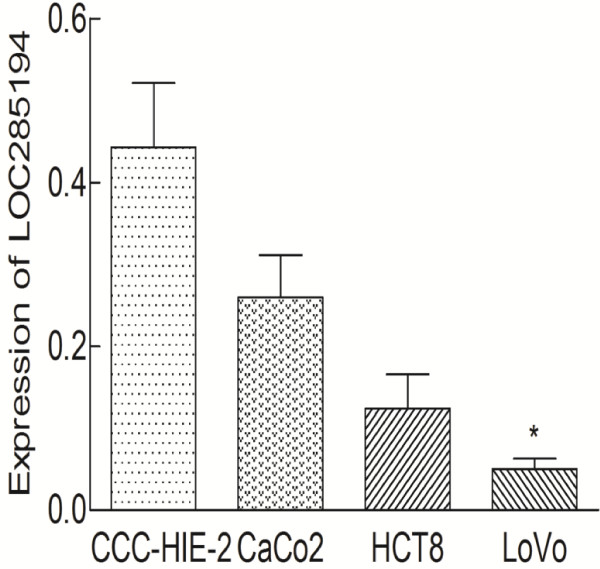
**Expression of LOC285194 in three colon cancer cell lines and normal intestinal mucous cell line.** LOC285194 expression levels were determined by quantitative real-time polymerase chain reaction in 3 colon cancer cell lines (CaCO-2, HCT8, and LoVo cells) and normal intestinal mucous cell line (CCC-HIE-2). Data are means ± SEM (n = 3). *Significantly different from control (p < 0.05).

RT-qPCR assays were further developed to quantify LOC285194 in colorectal cancer cell lines, including CaCO-2, HCT8, LoVo cells, and normal intestinal mucous cell line CCC-HIE-2. A significant low expression of LOC285194 was found in LoVo (p = 0.02) compared to CCC-HIE-2, but there was no significant difference for CaCO-2 (p = 0.127) and HCT8 (p = 0.075, Figure 2).

### LOC285194 expression and clinicopathologic factors in CRC

To assess the correlation of LOC285194 expression with clinicopathologic data, the expression levels of LOC285194 in tumor tissues were categorized as low or high in relation to the mean value. Clinicopathologic factors were analyzed in the high and low LOC285194 expression groups (Table 1). The low LOC285194 group (n = 48) showed larger tumor size, higher tumor stage and more distant metasatsis than the higher LOC285194 expression group (n = 33; p < 0.05). However, there was no significant correlation between LOC285194 expression and other clinicopathologic features, such as age, gender, tumor location, histologic grade, lymphatic metastasis, venous invasion or nervous invasion (p > 0.05; Table 1).

**Table 1:** Relationship between LOC285194 expression and clinicopathologic parameters of colorectal cancer patients

**Characteristics**	**Number of case**	**LOC285194 expression**				***p *value**
		**High (n = 33)**	**%**	**Low (n = 48)**	**%**	
Age (years)	81	55.2 ± 11.5		57.4 ± 11.3		0.225
Gender						0.339
Male	39	18	54.5	21	43.8	
Female	42	15	45.4	27	56.2	
Tumor size						0.015**
<4 cm	36	20	60.6	16	33.3	
≥4 cm	45	13	39.4	32	60.4	
Location						0.064
Colon	44	22	66.7	22	45.8	
Rectum	37	11	33.3	26	54.2	
Histologic grade						0.752
Well/moderately	58	23	69.7	35	72.9	
Poorly/others	23	10	30.3	13	27.1	
Depth of invasion						0.529
T1,T2	23	9	27.3	14	29.2	
T3,T4	58	24	72.7	34	70.8	
Lymphatic metastasis						0.052
Absent	46	23	69.7	23	47.9	
Present	35	10	30.3	25	52.1	
Venous invasion						0.209
Absent	55	25	75.8	30	62.5	
Present	26	8	24.2	18	37.5	
Nervous invasion						0.229
Absent	69	30	90.9	39	81.2	
Present	12	3	9.1	9	18.8	
Distant metastasis						0.046**
Absent	65	30	90.9	35	72.9	
Present	16	3	9.1	13	27.1	
Tumor stage*						0.034**
I and II	45	23	69.7	22	45.8	
III and IV	36	10	30.3	26	54.2	

* Tumor stage was obtained according to the TNM criteria. ** *p* < 0.05.

### Correlation between LOC285194 expression and prognosis of CRC patients

**Figure 3:**
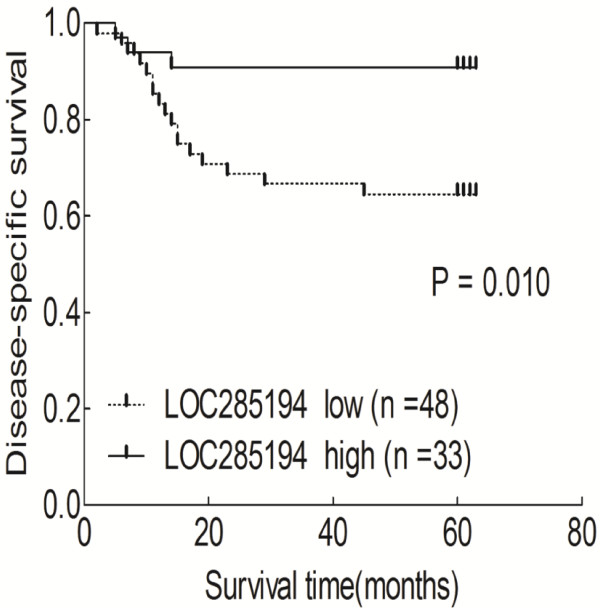
**Kaplan-Meier survival curves according to LOC285194 level.** Patients with low LOC285194 expression (n = 48) had a significantly poorer prognosis than those with high LOC285194 expression (n = 33, p = 0.010). P-value was calculated by Log-Rank test.

Disease-specific survival (DSS) curves were plotted according to LOC285194 expression level by the Kaplan-Meier method. During follow-up, 20 patients died of CRC. As shown in Figure 3, patients with low LOC285194 expression had a significantly poorer prognosis than those with high LOC285194 expression (p = 0.010). Univariate analysis of DSS revealed that the relative level of LOC285194 expression (p = 0.019), histologic grade (p = 0.002), tumor depth (p = 0.017), lymphatic metastasis (p = 0.001), venous invasion (p = 0.019), nervous invasion (p = 0.048), distant metastasis (p < 0.001), and tumor stage (p = 0.001) were prognostic indicators (Table 2). The other clinicopathological features, such as age, gender, tumor location, and tumor size, were not statistically significant prognosis factors (p > 0.05; Table 2). Variables with a value of p < 0.05 were selected for multivariate analysis. Multivariate analysis showed that LOC285194 expression was an independent prognostic indicator for DSS in patients with CRC (p = 0.034) in addition to presence of lymphatic metastasis (p = 0.017) and distant metastasis (p = 0.006) (Table 3).

**Table 2:** Univariate analysis of clinicopathological factors for disease-specific survival

**Variable**	**N**	**Hazard ratio**	**95% CI**	***p *value**
Age (years)				
<56	35	1		
≥56	46	0.754	0.314-1.812	0.528
Gender				
Male	39	1		
Female	42	0.734	0.304-1.772	0.492
Tumor size				
<4 cm	36	1		
≥4 cm	45	0.774	0.322-1.859	0.556
Location				
Colon	44	1		
Rectum	37	1.030	0.421-2.521	0.948
Histologic grade				
Well/moderately	58	1		
Poorly/others	23	4.020	1.662-9.726	0.002*
Depth of tumor				
T1,T2	23	1		
T3,T4	58	4.870	0.737-4.531	0.017*
Lymphatic metastasis				
Absent	46	1		
Present	35	3.664	1.009-6.587	0.001*
Venous invasion				
Absent	55	1		
Present	26	2.362	0.982-5.680	0.019*
Nervous invasion				
Absent	69	1		
Present	12	3.605	1.434-9.064	0.048*
Distant metastasis				
Absent	65	1		
Present	16	0.076	0.019-0.294	<0.001*
Tumor stage				
I and II	45	1		
III and IV	36	3.453	0.468-4.476	0.001*
LOC285194				
Low	48	1		
High	33	3.201	1.318-7.774	0.019*

N. number of patients, CI. confidence interval. * <0.05.

**Table 3:** Multivariate analysis of clinicopathological factors for disease-specific survival

**Variable**	**HR**	**95% CI**	***p *value**
Histologic grade (poor, others/well, mod)	1.649	0.566-4.801	0.348
Depth of tumor (T3,T4/T1,T2)	2.843	0.069-1.652	0.279
Lymphatic metastasis (present/absent)	1.138	0.001-9.942	0.017*
Venous invasion (present/absent)	0.726	0.215-2.445	0.605
Nervous invasion (present/absent)	0.674	0.184-2.469	0.551
Distant metastasis(present/absent)	2.076	1.642-8.802	0.006*
Tumor stage (III + IV/I + II)	1.520	0.006-5.374	0.156
LOC285194 (low/high)	0.337	0.083-4.367	0.034*

HR. Hazard ratio, CI. Confidence interval, well. well-differentiated, mod. moderately differentiated, poor. poorly differentiated, * < 0.05.

## Discussion

LncRNAs have been recently implicated as having oncogenic and tumor suppressor roles. One example of such an oncogenic lncRNA is Hox transcript antisense intergenic RNA (HOTAIR). HOTAIR expression was low in normal breast epithelia but high in primary breast cancer as well as metastatic lesions, HOTAIR expression level was a powerful predictor of patient outcomes [12]. In another study, HOTAIR expression levels were higher in cancerous tissues than in corresponding noncancerous tissues of stage IV CRC patients and those patients with high HOTAIR expression had a relatively poorer prognosis, linking a lncRNA with cancer invasiveness and patient prognosis [13]. Another classic oncogenic lncRNA is Metastasis-Associated Lung Adenocarcinoma Transcript 1 (MALAT-1), which was widely expressed in normal human tissues [14,15], but was upregulated in six other types of cancer [16-20]. In addition, increased expression of MALAT-1 has been recently shown to be an independent prognostic factor for HCC following liver transplantation [21] and non-small cell lung cancer [22].

Tumor-suppressor lncRNAs could phenotypically affect cells by promoting tumor-suppressor pathways, and when their function is compromised, cells are prone to develop cancer. In support of this notion, a few studies have elucidated several examples of ‘tumor-suppressor lncRNAs’. For example, Growth Arrest-Specific 5 (GAS5) has been observed to be downregulated in breast cancer, perhaps to sensitize cells to apoptosis by regulating the activity of glucocorticoids in response to nutrient starvation [23,24]. In addition, genetic aberrations at the GAS5 locus have been found in many types of tumors, including melanomas and breast and prostate cancers [25,26], though their functional significance has not yet been established. Another tumor-suppressor lncRNA is lincRNA-p21, which acts as a transcriptional repressor in the canonical p53 pathway and plays a role in triggering apoptosis [27]. These data demonstrated the potential tumor-suppressor role of lncRNAs; however, the relationship between tumor-suppressor lncRNA and cancer patient prognosis remains unclear.

Recently, Pasic and colleagues have characterized a region of chr3q13.31, designated osteo3q13.31, which harbors frequent focal copy number alterations (CNAs) and loss of heterozygosity (LOH) in primary osteosarcoma samples and cell lines [10]. They found that most osteo3q13.31 CNAs involve two ncRNAs, LOC285194 and BC040587 and, sometimes, the limbic system-associated membrane protein (LSAMP) tumor suppressor (TS). In addition, they showed the most effect of osteo3q13.31 CNAs on LOC285194 expression by RT-qPCR among 43 primary osteosarcoma tumor biopsies and 5 osteosarcoma cell lines. Interestingly, focal osteo3q13.31 biallelic deletions were common in cell lines from various cancers, including gastrointestinal tract. Given the potential roles of lncRNAs in tumor-suppressive pathways, we speculated that LOC285194 was also misregulated in CRC development and/or progression. This functional lncRNA may also link to prognosis of CRC patients. To support of these hypotheses, we firstly investigated whether LOC285194 is detectable and altered in 81 pairs of colorectal cancer tissues and adjacent normal tissues by RT-qPCR. Then, a potential relationship between this lncRNA levels in tumor tissues and existing clinicopathological features of CRC, such as tumor size, location, histologic stage, depth of invasion, the status of lymphatic metastasis, venous invasion, nervous invasion, distant metastasis and disease free survival, was investigated.

Using GAPDH as normalization control, we confirmed that LOC285194 expression was significantly lower in 81 tumor tissues compared with adjacent normal tissues (p < 0.001). Additionally, LOC285194 expression is markedly decreased in LoVo colorectal cancer cell lines compared with normal intestinal mucous cell line using RT-qPCR assays (p = 0.02). The low LOC285194 group showed larger tumor size, higher tumor stage and more distant metasatsis than the higher LOC285194 expression group. Moreover, we found that low LOC285194 expression patients had a significantly poorer prognosis than those with high LOC285194 expression, consistent with reports of tumor-suppressive role of LOC285194 in osteosarcoma [10].

The precise molecular mechanisms behind the altered expression of LOC285194 in CRC are unclear. In osteosarcoma, changes in expression of LOC285194 could be explained by CNAs at osteo3q13.31 [10]. Colorectal carcinogenesis is a complex, multistep and multifactorial event, whether low expression of LOC285194 was associated with CNAs at 3q13.31 in CRC remains to be elucidated. In addition, the effects of altered expression of LOC285194 in the development and/or progression of CRC are fascinating. Pasic and colleagues have demonstrated that depletion of LOC285194 can not only promote cell growth in MTT assays, but also increase the G1 population in cell cycle analysis. However, depletion of LOC285194 mRNA had no effect on osteoblast migration. This implicates LOC285194 as functional growth suppressor in osteoblasts in vitro. The way that lncRNA regulates the expression of its target genes is very complex. LOC285194 can act as growth suppressors through regulation of apoptotic and cell cycle transcripts and VEGF/VEGFR1 in osteoblasts [10]. Nevertheless, since it’s highly possible that target genes of lncRNA differ between specific tissues and cell types, specific target genes controlled by LOC285194 for CRC remained unknown, whether or not it also regulates VEGF/VEGFR1 and BCL2 will require detailed investigation. In future work, high-throughput techniques should be applied to obtain a global view on the changes of mRNA molecules and to elucidate the mechanisms of regulation of LOC285194.

## Conclusion

Our results showed that LOC285194 expression was significantly decreased in colorectal cancer tissues and cell lines. A lower expression of LOC285194 was detected in tumor of larger size, higher tumor stage and with distant metastasis. In addition, the downregulation expression of LOC285194 was associated with poor prognosis. These findings suggested that LOC285194 might be a novel prognostic indicator in CRC and may be a potential target for diagnosis and gene therapy.

## Competing interests

The authors declare that they have no competing interests.

## Authors’ contributions

PQ and MX performed experiments and were responsible for data collection, analysis, interpretation of the results, and writing the manuscript. SN and DH were responsible for conducting the data analysis in cooperation with PW and CT. XZ provided clinical samples for performance of experiments and validation of data. XD were responsible for experimental design, analysis and interpretation. All authors have read and approved the final manuscript.
